# Supplementary material for: Dietary Protein Levels Modulate the Antioxidant Capacity during Different Growth Stages in Huanjiang Mini-Pigs
Source: Antioxidants (Basel). 2023 Jan 7;12(1):148. doi: 10.3390/antiox12010148 (PMC9854851; doi:10.3390/antiox12010148)
Supplement: Supplementary file 1 [file antioxidants-12-00148-s001.zip › antioxidants-2136019-supplementary.pdf]

Supplementary Material

Table S1 Ingredients and composition of the basal diet for 5−10 kg body weight stage (air-dried, %)

Items	Dietary crude protein (CP) levels (%)				
	14	16	18	20	22
**Ingredients, %**					
Corn	61.20	56.20	51.80	47.50	42.70
Soybean meal (43.9%)	5.70	10.10	13.20	15.60	18.80
Fish meal	2.00	2.00	2.00	2.00	2.00
Wheat bran	9.00	9.00	9.00	9.00	9.00
Whey powder (85% lactose)	10.00	10.00	10.00	10.00	10.00
Extruded full-fat soybean	5.00	5.00	5.00	5.00	5.00
Soy protein concentrate	1.00	2.10	4.00	6.30	8.20
Corn oil	0.40	0.20			
L-Lys (98.5%)	1.10	0.90	0.75	0.55	0.40
DL-Met (99%)	0.20	0.15	0.15	0.10	0.10
L-Thr (99%)	0.40	0.35	0.25	0.20	0.10
L-Trp (99%)	0.10	0.10	0.05	0.05	
Dicalcium phosphate	1.40	1.40	1.30	1.20	1.20
Premix[1]	2.50	2.50	2.50	2.50	2.50
Total	100.00	100.00	100.00	100.00	100.00
**Nutrient levels[2]**					
Metabolic energy (MJ/kg)	13.73	13.69	13.68	13.72	13.74
CP	14.09	16.10	18.10	20.07	22.08
SID Lys	1.36	1.34	1.36	1.34	1.36
SID Met	0.39	0.37	0.39	0.37	0.40
SID Thr	0.76	0.79	0.77	0.80	0.77
SID Trp	0.21	0.24	0.22	0.24	0.22
Ca	0.79	0.80	0.80	0.79	0.80
Total phosphorus	0.66	0.69	0.69	0.70	0.72

[1]Provided the following per kg of the diet: Cu, 128 mg; Mn, 97.60 mg; Zn, 109 mg; Fe, 197.60 mg; Se, 1 mg; I, 1 mg; Co, 1 mg; VA, 32,500 IU; VD_3, 10,000 IU; VE, 80 IU; VK_3, 10 mg; VB_1, 10 mg/kg; VB_2, 25 mg; VB_6, 8 mg; VB_{12}, 0.075 mg; biotin, 0.075 mg; folic acid, 5 mg; nicotinamide, 100 mg; pantothenic acid, 50 mg; choline, 1,600 mg; limestone, 0.80%; NaCl, 0.30%; antimycotic, 0.10%; ethoxyquinoline (33%), 0.05%; acidifier, 0.25%.

[2]Calculated according to the nutrient requirements of swine (NRC, 2012); SID, standard ileal digestible.

Table S2 Ingredients and composition of the basal diet for 10–20 kg body weight stage (air-dried, %)

Items	Dietary crude protein (CP) levels (%)				
	12	14	16	18	20
**Ingredients, %**					
Corn	72.10	66.50	60.90	55.45	49.80
Soybean meal (43.9%)	0.50	6.75	12.95	19.00	25.25
Soybean meal (fermented)	4.00	4.00	4.00	4.00	4.00
Fish meal	2.00	2.00	2.00	2.00	2.00
Wheat bran	8.00	8.00	8.00	8.00	8.00
Whey powder (85% lactose)	5.00	5.00	5.00	5.00	5.00
Soybean oil	0.80	0.60	0.40	0.20	
L-Lys (98.5%)	1.10	0.95	0.75	0.60	0.40
DL-Met (99%)	0.20	0.15	0.15	0.10	0.10
L-Thr (99%)	0.45	0.35	0.30	0.20	0.10
L-Trp (99%)	0.15	0.10	0.05	0.05	
Dicalcium phosphate	1.20	1.10	1.00	0.90	0.85
Premix[1]	4.50	4.50	4.50	4.50	4.50
Total	100.00	100.00	100.00	100.00	100.00
**Nutrient Levels[2]**					
Metabolic energy (MJ/kg)	13.73	13.68	13.64	13.59	13.54
CP	12.06	14.08	16.10	18.08	20.08
SID Lys	1.22	1.24	1.22	1.24	1.23
SID Met	0.38	0.35	0.38	0.35	0.38
SID Thr	0.74	0.72	0.75	0.73	0.71
SID Trp	0.23	0.21	0.19	0.22	0.20
Ca	0.70	0.70	0.70	0.69	0.70
Total phosphorus	0.59	0.60	0.61	0.61	0.63

[1]Provided the following per kg of the diet: Cu, 128 mg; Mn, 97.6 mg; Zn, 109 mg; Fe, 197.6 mg; Se, 1 mg; I, 1 mg; Co, 1 mg; VA, 32,500 IU; VD_3, 10,000 IU; VE, 80 IU; VK_3, 10 mg; VB_1, 10 mg/kg; VB_2, 25 mg; VB_6, 8 mg; VB_{12}, 0.075 mg; biotin, 0.075 mg; folic acid, 5 mg; nicotinamide, 100 mg; pantothenic acid, 50 mg; choline, 1,600 mg; limestone, 0.80%; sucrose, 2%; NaCl, 0.3%; antimycotic, 0.10%; ethoxyquinoline (33%), 0.05%; acidifier, 0.25%.

[2]Calculated according to the nutrient requirements of swine (NRC, 2012); SID, standard ileal digestible.

Table S3 Ingredients and composition of the basal diet for 20–30 kg body weight stage (air-dried, %)

Items	Dietary crude protein (CP) levels (%)				
	**10**	**12**	**14**	**16**	**18**
**Ingredients, %**					
Corn	69.65	64.25	58.65	53.35	47.60
Soybean meal (43.9%)	1.00	6.90	13.20	19.10	25.50
Soybean meal (fermented)	2.00	2.00	2.00	2.00	2.00
Wheat bran	12.00	12.00	12.00	12.00	12.00
Rice hull and bran	5.00	5.00	5.00	5.00	5.00
Soybean oil	3.00	2.80	2.60	2.30	2.10
L-Lys (98.5%)	0.95	0.80	0.60	0.45	0.25
DL-Met (99%)	0.15	0.15	0.10	0.10	0.05
L-Thr (99%)	0.35	0.30	0.20	0.15	0.05
L-Trp (99%)	0.10	0.10	0.05	0.05	
Dicalcium phosphate	1.10	1.00	0.90	0.80	0.75
Premix[1]	4.70	4.70	4.70	4.70	4.70
Total	100.00	100.00	100.00	100.00	100.00
**Nutrient levels[2]**					
Metabolic energy (MJ/kg)	13.27	13.23	13.18	13.12	13.06
CP	10.12	12.11	14.10	16.10	18.13
SID Lys	1.00	1.01	1.00	1.01	1.00
SID Met	0.29	0.31	0.29	0.31	0.29
SID Thr	0.59	0.61	0.59	0.62	0.60
SID Trp	0.17	0.20	0.18	0.21	0.19
Ca	0.67	0.67	0.66	0.66	0.67
Total phosphorus	0.54	0.54	0.55	0.56	0.57

[1]Provided the following per kg of the diet: Cu, 128 mg; Mn, 97.6 mg; Zn, 109 mg; Fe, 197.6 mg; Se, 1 mg; I, 1 mg; Co, 1 mg; VA, 32,500 IU; VD_3, 10,000 IU; VE, 80 IU; VK_3, 10 mg; VB_1, 10 mg/kg; VB_2, 25 mg; VB_6, 8 mg; VB_{12}, 0.075 mg; biotin, 0.075 mg; folic acid, 5 mg; nicotinamide, 100 mg; pantothenic acid, 50 mg; choline, 1,600 mg; limestone, 1%; sucrose, 2%; NaCl, 0.3%; antimycotic, 0.10%; ethoxyquinoline (33%), 0.05%; acidifier, 0.25%.

[2]Calculated according to the nutrient requirements of swine (NRC, 2012); SID, standard ileal digestible.

Table S4 Primer sequences for intestinal mucosa mRNA analysis

Genes	Primers (5‘-3’)	Amplicon Size (bp)	Accession NO.
β-Actin	F: GATCTGGCACCACACCTTCTACAAC<br>R: TCATCTTCTCACGGTTGGCTTTGG	107	XM_021086047.1
CAT	F: AGCCTACGTCCTGAGTCTCTGC<br>R: TCCATATCCGTTCATGTGCCTGTG	90	NM_214301.2
CuZnSOD	F: CCAGTGCAGGTCCTCACTTCAATC<br>R: CGGCCAATGATGGAATGGTCTCC	172	NM_001190422.1
GPx-1	F: TGCTCATTGAGAACGTAGCGT<br>R: CAGGATCTCCCCATTCTTGGC	161	NM_214201.1
GPx-4	F: GATTCTGGCCTTCCCTTGC<br>R: TCCCCTTGGGCTGGACTTT	173	NM_214407.1
KEAP-1	F: CGCCTCATCGAGTTCGCTTACAC<br>R: GCACGGACCACACTGTCAATCTG	107	NM_001114671.1
MnSOD2	F: GGACAAATCTGAGCCCTAACG<br>R: CCTTGTTGAAACCGAGCC	159	NM_214127.2
NQO-1	F: GTGGAAGCCGCAGACCTTGTG<br>R: CGTTCAAACCAGCCTTTCAGAATAGC	83	NM_001159613.1
Nrf-1	F: CGATGCTTCAGAATTGCCAACTACAG<br>R: GCGTTGTCTGGATGGTCATCTCAC	125	XM_021078993.1
Nrf-2	F: CCAATTCAGCCAGCACAACACATC<br>R: GACTGAGCCTGGTTAGGAGCAATG	149	XM_003133500

CAT, catalase; *GPX*, Glutathione peroxidase; *KEAP*, Kelch like ECH associated protein; *NQO1*, quinone oxidoreductase 1; *Nrf*, nuclear respiratory factor.
